# Myocardial Perfusion Spect Imaging in Dextrocardia: A Case Report

**DOI:** 10.4274/Mirt.151

**Published:** 2013-08-01

**Authors:** Semra Özdemir, Emine Gazi

**Affiliations:** 1 On Sekiz Mart University Faculty of Medicine, Department of Nuclear Medicine, Çanakkale, Turkey; 2 State of Hospital , Department of Cardiology, Çanakkale, Turkey

**Keywords:** Dextrocardia, myocardial perfusion imaging

## Abstract

The myocardial perfusion scintigraphy acquisition and analysis present some technical differences in the rare dextrocardia cases. Here we report a case of a 38 year-old woman with dextrocardia who had been applied myocardial perfusion scintigraphy. Presented case showed that the thoracic and abdominal organs had a mirror image with situs inversus totalis type dextrocardia. The incidence of coronary heart disease and life span of people with situs inversus totalis are the same as the normal population. So we may apply myocardial perfusion scintigraphy to this patient group. The current case is presented in order to remind the special applications of myocardial perfusion SPECT imaging in patients with dextrocardia.

**Conflict of interest:**None declared.

## INTRODUCTION

Dextrocardia is a rare cardiac malposition. The position of the heart in the thorax determines the orientation of the long axis of the apex (base-to-apex axis). In levocardia the base-to-apex axis of the heart is pointed towards the left, in dextrocardia towards the right and in mesocardia it is pointed vertically (1,2,3,4,5). Cardiac situs is determined by the atrial location in the thorax and there are 3 types: 1) Situs solitus: right atrium on the right, left atrium on the left, 2) Situs inversus: right atrium on the left, left atrium on the right. 3) Situs ambiguus: atrial isomerism (6). Situs solitus is the normal position, and situs inversus is the mirror image of situs solitus. Dextrocardia can be seen with situs solitus or situs inversus. Isolated dextrocardia is also termed situs solitus with dextrocardia. The cardiac apex points to the right, but the viscera are otherwise in their usual positions. Situs inversus with dextrocardia is also termed situs inversus totalis, a congenital anomaly that is characterized by the thoracic and abdominal organs are located in mirror position in the opposite side of the body (7,8,9,10,11). Dextrocardia associated to situs inversus totalis is a rare congenital condition (prevalence of 1:10000) (12).

In this report, we present the case of a 38 year-old woman with dextrocardia associated with situs inversus totalis who was admitted to our clinic for myocardial perfusion imaging. 

## CASE REPORT

**Figure 1:**
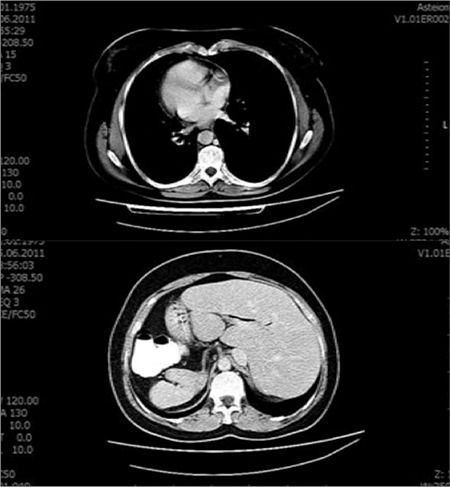
Computed tomography shows dextrocardia with situs solitus inversus (the heart on the right, the liver on the left side and spleen on the right side).

A 38 year-old woman who was suffering from chest pain and shortness of breath was admitted to our clinic in order to investigate myocardial ischemia. The patient was informed about myocardial perfusion scintigraphy (MPS) and she signed the consent form which is obtained for all applications. In the previous interview the patient informed us her thoracic and abdominal organ asymmetry. Situs inversus totalis was also reported by computed tomography in the patient (Figure 1). 

Dipyridamole exercise and one day rest-stress protocol was used for the 99mTc sestamibi myocardial perfusion gated single photon emission computed tomography (SPECT). SPECT imaging was realized by a dual head gamma camera (General Electric Infinia) using 64x64 matrix and 30 projections (rest:25s, stres:20s). For ECG-gated study camera acquisition was triggered to R-wave, 8 frames collected per R-R interval. 

Two different acquisitions and analyses were performed to understand the difference in dextrocardia in the current case. 

**Figure 2:**
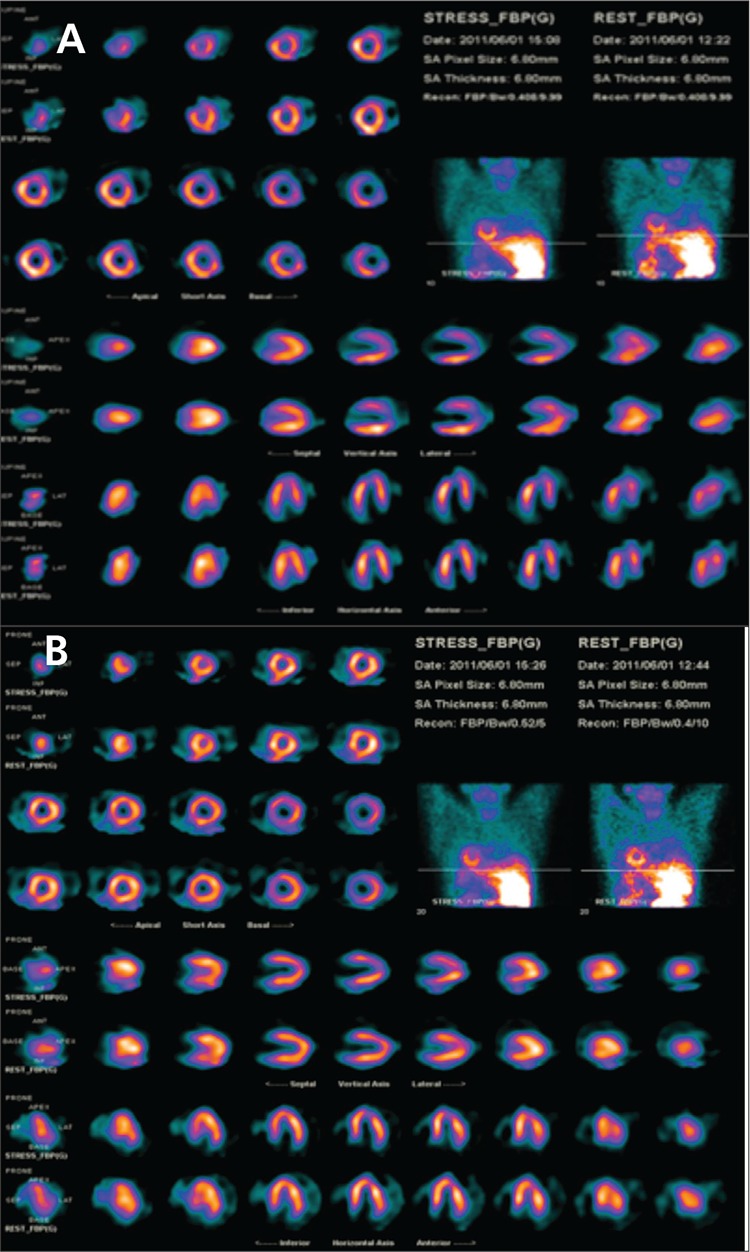
A) Short-axis, vertical long-axis and horizontal long-axis slices and raw data of stress-rest myocardial perfusion SPECT in the normal acqusition and analysis of dextrocardia patient (the septal and lateral walls change locations cause false positive lateral wall defect.). B) Short-axis, vertical long-axis and horizontal long-axis slices and raw data of myocardial perfusion SPECT in the dextrocardia appopriate acqusition and analysis of dextrocardia patient (Lateral and septal walls are normal

The first MPS was performed according to our clinical routine protocols. SPECT procedure was carried out by using a 180 degrees counterclockwise circular orbit, beginning at 45-degree right anterior oblique projection and ending at 45-degree left posterior oblique projection in head out (Feet First Supine) position. The first raw images were reconstructed by using normal analysis parameters (Figure 2 A). 

The second MPS was performed according to dextrocardia protocols. Briefly, SPECT procedure was carried out by using a 180 degrees counterclockwise circular orbit, beginning at 45-degree left anterior oblique projection and ending at 45-degree right posterior oblique projection in head in (Head First Supine) position. The second raw images were reconstructed by using dextrocardia analysis parameters of camera software. Thus before the projection image was selected for the analysis, in the normal myocardial perfusion analysis program the patient was positioned to Feet First Prone although the patient was positioned to Head First Supine during imaging, Feet First Prone position was selected while doing the analysis so that the heart and spatial position of the patient with dextrocardia was positioned like a normal patient. Then the raw data was selected and the normal analysis steps were followed. (Figure 2 B). 

When the two images were evaluated it can be stated that in the first imaging because the lateral and septal walls changed positions, perfusion defect was observed in basal sections of lateral walls in both stress and rest images while in the second imaging, perfusion was in normal ranges on all the walls. On the other hand, in the first gated imaging ejection fraction was measured as 60% and mild wall motion and thickening abnormalities were observed on anterolateral and inferolateral walls. However, in the second gated imaging EF was measured as 70% and no wall motion or thickening abnormalities were observed. Eventually the myocardial perfusion gated SPECT results of the current patient were reported as in the normal range. 

Literature Review and Discussion

MPS is a non-invasive imaging modality which is commonly used to evaluate the left ventricular wall supply in cardiovascular disease. Although the dextrocardia is a rare clinical situation, myocardial perfusion imaging SPECT can be applied to this patient group in nuclear medicine. Yet dextrocardia patients with situs inversus totalis have the same incidence of coronary artery disease and life expectancy compared to the general population (12). In the literature there are a few reports about myocardial perfusion scintigraphy with dextrocardia. Turgut et al reported a case of mirror-image dextrocardia and constant complete left bundle branch block with false positive Tl-201 SPECT findings (13). Slart et al have also reported ananother case of dextrocardia in whom they used routine myocardial perfusion images because of the rotational direction of the perpendiculary positioned camera heads was fixed and adaptation to a right positioned heart was not possible. Therefore they corrected emission scans for attenuation by a transmission scan during reconstruction (14). 

In situs inversus totalis, the heart is located on the left side of thorax and has a mirror image of normal anatomical position. In this situation the anterior and posterior walls of the heart do not change places, but localization of the septal and lateral walls do change in right-left direction. Knowing the exact position of the heart in dextrocardia cases has a crucial role for a valid study. So, it is necessary to know how to perform the acquisition and analysis of myocardial perfusion imaging in patients with dextrocardia. Before myocardial perfusion imaging is carried out, it must be assured that the gamma camera software used in the process has a special acquisition and analysis protocol for dextrocardia. Even the acquisition performed is based on anatomical differences in dextrocardia, it may cause some problems at the analysis of data and the images may not be accepted to be analyzed. Although some of the gamma camera software makes it possible to acquire data in the patient with dextrocardia, it may not allow the users to process the data. For this reason, when dextrocardia is diagnosed in a patient, the acquisition and analysis protocol must be questioned both before and during monitoring. If there is a recommended protocol for the software of the camera, acquisition and analysis have to be done according to that protocol. 

If the patient with dextrocardia is not well recognized and the normal acquisition-analysis protocol is applied, it causes a significant errors. In dextrocardia, the anterior and posterior walls of the heart does not change place but the septal and lateral walls, change locations. The altered orientation results in an apparent defect in a normal lateral wall (Figure 2 A). Even if the patient had normal perfusion in left ventricular wall, lateral wall perfusion defect can be reported. As in our case, that wall is actually the septum, and the perfusion is normal (Figure 2 B). So, it is important to review the raw data and examine whole data set of myocardial perfusion SPECT images. In such cases SPECT-CT enables a direct correlation of anatomic information and functional information, resulting in better localization and definition of scintigraphic findings.

In conclusion, although it is a rare case, the patient group with dextrocardia may be referred for the evaluation of myocardial perfusion imaging in nuclear medicine practice. For this reason, the presented case emphasizes the fact that it is important to be well prepared for an accurate myocardial perfusion imaging, analysis and reporting of dextrocardia. Before myocardial perfusion imaging is carried out, it must be assured that the gamma camera software used in the process has a special acquisition and analysis protocol for dextrocardia.
